# Observations on Sudden Deaths Occasioned by a Rupture of the Left Ventricle of the Heart

**Published:** 1788

**Authors:** M. Portal

**Affiliations:** Professor of Anatomy, and Member of the Royal Academy of Sciences at Paris, &c.


V. *Obſervations on ſudden Deaths occaſioned by a Rupture of the left Ventricle of the Heart. By M.* Portal, *Profeſſor of Anatomy, and Member of the Royal Academy of Sciences at Paris, &c. Tranſlated from the French* *.

IT would perhaps hardly be believed, were it not demonſtrated by experience, that a muſcular organ, like the heart, the parietes of which are ſo firm, is capable of being ruptured, and thus occaſioning the moſt ſudden death. Harvey has related an inſtance of this kind in his immortal work on the circulation of the blood,

* This article is extracted from the Memoirs of the Royal Academy of Sciences at Paris, for the year 1784, publiſhed in 1787.

and and ſucceeding anatomiſts have mentioned other ſimilar facts in their writings.

It will be ſufficient to read the works of Morgagni, Senac, and Lieutaud, to be convinced that the auricles and ventricles of the heart have occaſioned, by their rupture, an extravaſation of blood within the pericardium; but the moſt extraordinary circumſtance in caſes of this ſort is, that it is not in the auricles, the texture of which is extremely thin, but in the ventricles, whoſe parietes are of ſo much thickneſs and ſtrength, that accidents of this ſort the moſt frequently occur. It ſeems to be certain alſo, that theſe ruptures happen more commonly in the left ventricle than in the right, although the parietes of the latter appear to be very yielding when compared with thoſe of the left ventricle. This fact is confirmed by my own obſervations, and by different caſes collected by the writers I have conſulted; and it is very different from the caſe of ſimple dilatations, which are more common in the auricles than in the ventricles; in the ſame manner as it is more uſual to ſee the auricles dilated beyond their natural diameter than the arteries, which laſt, on the contrary, give way more frequently than the veins.

Anatomical

Anatomical writers have given an account of all these varieties; but as they do not seem to have paid sufficient attention to ruptures of the left ventricle, and to the causes that produce them, I have thought it right to communicate the observations I have had an opportunity of making on this subject. They are, perhaps, not altogether new; but they will at least serve to corroborate the facts of the same kind related by different writers, and particularly by Morgagni, Senac, Haller, and Lieutaud.

It was in 1768 that I had occasion to make the first observation of this kind in the case of Madame de Chabanes, of the *rue des Jeuneurs*, who, till the age of sixty years, had enjoyed pretty good health. She then began to complain of a difficulty of breathing, which gradually increased, and at length became very distressing whenever she walked up stairs, or got into a carriage. Her pulse at such times was very irregular and even intermittent, and was some time before it returned to its natural state. It was remarkable, that she used to find herself less affected in a rough carriage than in such as were better suspended. She lived with this complaint till about the age of sixty-five years; and as she found that bleeding was the remedy remedy from which ſhe experienced the moſt relief, ſhe had accuſtomed herſelf to have recourſe to it twice or thrice every year, and ſometimes oftener: but the ſlighteſt agitation of mind conſtantly brought on a palpitation of the heart; and at length one day, happening to be in violent anger, the palpitation became ſtronger than ever; ſhe was able to breathe only with extreme difficulty; her face became pale, and ſhe died ſuddenly.

I aſſiſted at the opening of the dead body with M. M. de Vernage and Malouin, who had been her phyſicians, and here is an account of what we obſerved.

In the lower belly there was an extravaſation, not very conſiderable in quantity, of a reddiſh ſerum; the liver was very hard and obſtructed; the gall bladder contained four calculi, and was contracted, having the form of a canal of nearly equal diameter through its whole length. Theſe were the only remarkable appearances in the abdominal cavity.

It was in the cheſt that we diſcovered the cauſes of death. The pericardium was found to be greatly diſtended with blood, much of which flowed out when the pericardium was opened. The auricles of the heart were of an enormous enormous ſize, and the right ventricle was at leaſt twice as large as the left; but their parietes, inſtead of being found thinner, as might have been expected from this diſtention, appeared, on the contrary, to be thickened. Their texture, however, was leſs firm than uſual, and they were ruptured in ſeveral places. Even the left ventricle, notwithſtanding the great thickneſs of its parietes, was torn in three places. One of theſe openings was at the anterior part of the heart near its baſis, and cloſe to the tendinous ſubſtance that connects the aorta with the heart; the other two were in the body of the ventricle, nearly parallel to that juſt now deſcribed, and a finger's breadth diſtant from it. The right ventricle was ruptured near the baſis, towards the upper edge of the ſeptum cordis.

The ſigmoidal valves of the aorta were hardened, and beſet with bony concretions. A collection of ſimilar concretions, placed immediately behind the former, ſo impeded the action of theſe valves as to allow only a very limited paſſage to the blood; and this paſſage was ſtill more obſtructed by the oſſifications with which the aorta itſelf was incruſted.

The pulmonary artery was oſſified in different places near its orifice in the right ventricle.

The

The ligamentous annular ſubſtance that unites it with the ventricle, and ſupports the valves, was very hard and unequal, and ſo enlarged, that the opening was ſingularly contracted. The valves had acquired the conſiſtence of cartilage; and the tubercles of Vidus Vidius, or, if you will, of Arantius, were as large as a pea, which is at leaſt ſeven or eight times larger than they are naturally. I have ſaid the tubercles of Vidius, becauſe he appears to have been acquainted with them before Arantius, to whom Morgagni has attributed the honour of the diſcovery. On this ſubject the reader may conſult my Hiſtory of Anatomy.

It was, without doubt, by theſe obſtacles that the heart was prevented from emptying itſelf in the ſyſtole. The reſiſtance the blood made to the contraction of the ventricles occaſioned theſe gradually to dilate; and, in the mean time, the circulation through the coronary veſſels being ſlackened, the blood they contained was extravaſated between the fibres of the heart; the texture of theſe fibres was weakened, and at length they were torn by their own contractions.

In ſome ſubjects who have died ſuddenly in conſequence of a rupture of the heart, and of whoſe caſes an account is given by Morgagni, there there have been found appearances in that organ nearly ſimilar to thoſe I have been deſcribing; but I am able to recollect no inſtance of a rupture of the two ventricles in the ſame ſubject, neither do I know that the tubercles of the valves have ever been found ſo much enlarged as in the caſe I have juſt now related. Without doubt the extreme ſoftneſs of the parietes of the heart, in this inſtance, might facilitate their being ruptured; but the ultimate cauſe of this accident muſt have been the preſſure made by the blood, on thoſe parts, in conſequence of its not being able to flow out freely.

The ſecond caſe I ſhall relate is, that of the Counteſs de Nevron, a lady who was extremely corpulent, and who had long been ſubject to a difficulty of breathing whenever ſhe uſed any exerciſe that was at all fatiguing. She came from Nancy to Paris without reſting by the way, and on the evening of her arrival experienced great difficulty of breathing, together with ſevere colicky pains. A phyſician, who was called to her aſſiſtance, found her pulſe very full, but remarkably irregular. About midnight ſhe complained that her difficulty of breathing was become much more diſtreſſing, and ſhe died almoſt at that inſtant.

Her

Her body preferved its warmth for a confiderable time after death, efpecially about that part of the cheft that correfponds with the heart; and on this account the diffection was delayed longer than ufual.

The body was fo loaded with fat, that its bulk was enormous. Under the integuments this fubftance was more than four fingers breadth in thicknefs; but the mufcles were of their natural firmnefs. The quantity of fat contained in the omentum was fo great, that it filled the greater part of the abdominal cavity. There was alfo much of it between the laminæ of the mefentery and around the kidnies. The liver was rather larger than ufual, and there were five calculi in the gall bladder.

There was fo great a quantity of fat between the laminæ of the mediaftinum, that they were confiderably feparated from each other, and of courfe the cavity of the cheft was diminifhed. The heart was covered with a layer of fat of more than two fingers breadth in thicknefs, and the pericardium was diftended with blood extravafated from an opening at the bafis of the heart near the aorta. The ligamentous fubftance that connects this artery with the heart was torn from the latter at its anterior part, fo as to occafion an opening, into which I could with eafe introduce my little finger. In other refpects the fubftance of the heart was as firm and compact as ufual. Neither of the ventricles, not even that which was torn, was at all larger than common; and there was no appearance of erofion in any part of their fubftance. No alteration was perceptible in the coronary veffels, fo that we could not attribute this accident to any defect in them, or to any preternatural affection of the mufcular fibres of the heart.

Notwithftanding the great quantity of blood extravafated within the pericardium, there was ftill much remaining both in the arteries and veins of the heart; and is it not to an excefs of this fluid, or to the enormous quantity of fat, that we may attribute the caufe of the rupture in this cafe?

The part at which this rupture took place deferves to be confidered. It was not at the apex of the heart, which is the thinneft part of that vifcus, and where (according to Morgagni and Senac) ruptures of this fort moft commonly happen; but at the bafis, and confequently at a part where the heart, from its tendinous ftructure, feems to be the ftrongeft.

The ventricle of the heart in which this preternatural opening took place, was, as we have ſeen, not more dilated than it is uſually; neither were its parietes ſoftened in the manner Senac and Morgagni found them to be in hearts covered with fat, and which were ruptured; ſo that this caſe forms an exception to what they have advanced on this ſubject: for thoſe celebrated anatomiſts have endeavoured to attribute the prediſpoſing cauſe of ruptures of the heart to ſome alteration in its texture. They imagined that the fat, collected about this viſcus, ſoftened its fibres, and was the cauſe why the blood, by diſtending it during the diaſtole, terminated by rupturing it. In ſome caſes alſo they have aſcribed this accident to an ulcer, which, by eroding the ſubſtance of the heart, had rendered it gradually thinner, till it could no longer reſiſt the impulſe of the blood; or they ſuppoſed that the ulcer had terminated by making a complete opening in the heart, and that this had occaſioned an eruption of blood into the pericardium.

All theſe cauſes have taken place, and their exiſtence has been confirmed by facts which the two writers juſt now mentioned have related and learnedly diſcuſſed; but in the caſe I have been deſcribing there was neither any apparent alteration ration of texture in the heart, nor any marks of ulceration: on the contrary, the powers of this viſcus ſeem to have been undiminiſhed, and it appears to have been leſs owing to a want of firmneſs in the parietes of the heart than to an increaſe of reſiſtance which they could not overcome, that the rupture took place; and this accident muſt have happened at the time of the ſyſtole or contraction of the heart.

It cannot reaſonably be allowed that the rupture of the heart takes place during the diaſtole, in the manner Senac and Morgagni have ſuppoſed, without admitting, as they have, a preternatural extenſion of the fibres of the ventricle previouſly to the rupture, by which means its capacity is more or leſs increaſed; but, as in the caſe I have laſt deſcribed, as well as in ſome others I could mention, the ventricle in which the rupture took place was not enlarged, we may venture, I think, to conclude that it did not happen in conſequence of any violent extenſion of the fibres of the heart. If their opinion was well founded, the heart would always burſt at that part of the ventricles where the ſubſtance of the heart, between ſome of the muſcular faſciculi, is as thin as the fineſt membrane; but the facts I have related demonſtrate the the contrary. All these fasciculi are drawn strongly towards each other during the contraction of the heart; the spaces they leave during the dilatation of that viscus then disappear, and they form a substance of infinitely greater firmness than that which is seen in the dead body: hence it seldom happens that the heart is torn in those places, as will appear from my cases, and from those which have been collected by anatomical writers. The ruptures that sometimes happen in other muscles serve to corroborate my opinion on this subject, as these have been known to give way in consequence of violent convulsions or excessive contractions, several memorable instances of which are quoted by Haller. Moreover, it may be asked by what means could the blood be driven into the ventricles with sufficient force to burst their parietes? This could only be from the venæ cavæ and auricles; but as the texture of these is infinitely weaker than that of the ventricles, we cannot ascribe to them such an effect, unless we suppose the texture of the ventricles to have been extremely weakened, which was not the case in the instance just now related.

A lady, sixty-five years old, thin, and of extreme sensibility, who had long been subject to palpitation palpitation of the heart which had been thought to be ſpaſmodic, had accuſtomed herſelf much to warm bathing. At length ſomebody adviſed her to try the cold in preference to the warm bath, and even to add ice, and apply ſome of it, in a large bladder, to her head. She accordingly adopted this method, and perſiſted in it even in very cold weather, till one day ſhe fell into a ſtate of ſyncope in the bath, and was taken out dead.

On opening the body, the pericardium was found to be ſo much dilated that the left lobe of the lungs was compreſſed by it, and puſhed up towards the upper part of the cheſt. This dilatation of the pericardium was occaſioned by extravaſated blood, partly in a fluid, and partly in a coagulated ſtate. A conſiderable coagulum was found adhering to the upper and poſterior part of the heart, and when this was removed we diſcovered an opening, of about eight tenths of an inch in length, that led to the left ventricle which was full of blood. The diameter of the aorta, at its origin, was much contracted; the valves were of the conſiſtence of cartilage, and turned backwards towards the heart; two of the fleſhy columns were torn, and ſome of their tendinous extremities mities were found adhering to the edges of the rupture. Theſe edges were jagged and irregular, ſomewhat reſembling a piece of cloth that has been torn with violence; but there was no appearance of ulceration, and the parietes of the heart were nearly of their natural firmneſs, if we except a ſmall ſpot cloſe to the rupture, where the ſubſtance was ſo extremely thin, that it ſeemed remarkable that the perforation did not extend to this part. The left ventricle, the pulmonary veſſels, and the right ventricle were much dilated, and the parietes of the latter were extremely thin. In the pulmonary artery and its valves there was nothing preternatural.

In this caſe the oſſifications that were formed about the orifice of the aorta, in the left ventricle, were, without doubt, the primary cauſe of the rupture of the heart; for by leſſening the diameter of that artery they muſt have impeded the paſſage of the blood through it, and the left auricle being prevented from emptying itſelf with ſufficient facility, the pulmonary veins and artery became too much diſtended. The conſequence of this was a dilatation of thoſe veſſels and of the cavities of the heart; and, as the parietes of the right ventricle are much weaker than thofe of the left, the former yielded and, of courfe, became more dilated than the latter, which, on the other hand, were the firft to become ruptured.

The application of cold feems, in this cafe, to have occafioned a confiderable determination of blood to the interior parts of the body. Hence the heart became overloaded, and the left ventricle, unable to free itfelf by its contractions, at length gave way. In other cafes we have feen a dilatation of the right ventricle occafioned by offification of the valves of the aorta, or by other obftacles to the paffage of the blood from the left ventricle; which fhews that the caufes of the alterations we find in the right fide of the heart are fometimes to be fought for in the left. But the cafe is different with refpect to dilatations of the left ventricle of the heart, the caufes to which they are owing having uniformly their feat in that part, and being unconnected with any changes in the right ventricle. I have repeatedly feen a dilatation of the latter ventricle in fubjects in whom the left ventricle, fo far from being enlarged, was even fmaller than ufual; and, in all fuch cafes, the caufe of this has appeared to be owing to an impediment to the circulation lation in the pulmonary artery. As an inftance of this kind I will juft mention a cafe that occurred to me about fifteen years ago. The fubject of it (M. Maffon of the rue Mazarine), conftantly fpent a great part of the day in blowing the french-horn. At length he began to be troubled with a difficulty of breathing, and hæmoptoe, for which he had recourfe to different remedies, and he was advifed to lay afide his french-horn; but he no fooner got a little better then he returned again to the ufe of it. Soon after this he began to complain of a palpitation of the heart, which, though at firft flight, foon became more violent and durable. He was troubled, about the fame time, with a continual fenfe of fuffocation, and alfo with pain about the fternum. His difficulty of breathing increafed, his pulfe became extremely irregular, and his legs fwelled. At this period he found that he breathed beft when he was in a ftanding pofture, or fitting on a high chair. It was obferved, however, that when he laid down, he experienced fome relief when his breaft was repeatedly and flightly compreffed; but notwithftanding this, he was unable to remain long in this pofition. He was fubject to frequent fainting, and at length died in a kind of suffocation. His extremities, during the last two days of his life, were as cold as marble.

The dissection shewed that his death had been occasioned by an enormous dilatation of the right ventricle of the heart, which was full of coagulated blood. In this case the blood had been unable to pass out of the right ventricle with so much ease and in the same quantity as it entered by the corresponding auricle, and this gave rise to a gradual enlargement of the ventricle; while, on the other hand, the left ventricle became contracted, a circumstance that uniformly takes place whenever the dilatation of the right ventricle is occasioned by any impediment in the orifice or course of the pulmonary artery.

Such are the facts I proposed to myself to communicate relative to this subject. They prove,

First, That ruptures of the left ventricle of the heart are more frequent then is generally imagined:

Secondly, That accidents of this sort may take place without any alteration by which the texture of the heart has been previously weakened:

Thirdly,

Thirdly, That they are oftentimes an effect of the contraction, and not of the dilatation of the heart produced by the influx of the blood:

Fourthly, That, in general, when the left ventricle is dilated, the right ventricle is so also; but that, on the contrary, the right ventricle is often dilated, while the left, instead of being likewise inlarged, is, in such cases, oftentimes contracted.

The greater part of these remarks having appeared to me to be interesting, I have been induced to give them a place in the present paper; and I have done this the more readily from a persuasion, that if it be useful to investigate the means nature employs for her preservation, it must be not less so to learn how she tends to her destruction, as well for the sake of enabling us to avail ourselves of suitable remedies when they can be employed with advantage, as to know when to abstain from active modes of treatments, in cases where they may not only be useless, but even dangerous.

To my own observations I shall here add some remarks on the rupture of the left auricle and ventricle of the heart communicated to me by M. Chaussier, an experienced surgeon at Dijon,

Dijon, and which are highly deferving attention.

In the month of November, 1769, M. Chauffier was called upon to examine the body of a young and ftout labouring man, named Stephen Grappin, at a place called Saulon. This perfon, who had been driving a waggon loaded with ftones, having mounted one of the horfes, fell down, and one of the wheels paffed flowly, and in an oblique direction, over the left clavicle and fide of the cheft, leaving him without any appearance of life.

After removing the integuments and mufcles, M. Chauffier found the articulation of the clavicle with the fternum injured; and a feries of fractures along the whole of the left fide of the thorax, extending obliquely from the anterior to the pofterior part.

The firft rib was fractured near the fternum; but this fracture was incomplete, for at the external furface there were ftill fome bony lamellæ that preferved the continuity of this rib, but fo that it clearly appeared to have undergone a gradual and violent compreffion. The fecond rib was fractured more obliquely outwards, and there were in the the body of this rib two fractures, at the diſtance nearly of three inches from each other. The reſt of the true ribs and the firſt of the falſe ribs were likewiſe each of them broken in two places; but the ſecond of the falſe ribs was ſo only in one.

There was no appearance of contuſion on the integuments, neither was there any extravaſation of blood in the cellular membrane; the pleura and lungs were free from injury; but the pericardium was greatly diſtended, and when opened, was found full of coagulated blood, the auricle being torn at its baſis near the ventricle, forming an opening large enough to admit two fingers to be introduced through it into the left ventricle.

M. Chauſſier thinks it evident that the rupture of the left auricle, in this caſe, was occaſioned by the preſſure on the curvature of the aorta; and, indeed, this can hardly be doubted when we conſider the direction of the wheel, the ſtate of the clavicle and the injury done to its articulation with the ſternum, the weight of the waggon, and the ſlowneſs of its motion. It would ſeem that, during the paſſage of the wheel over the cheſt, the curvature of the aorta being compreſſed, the paſſage of the blood blood through it was impeded, and the left ventricle becoming, of courſe, preternaturally diſtended, the contractile power of the heart augmented in proportion to the reſiſtance it experienced, and the conſequence of all this was a rupture in the weakeſt part of the auricle; in the ſame manner, adds M. Chauſſier, as we ſometimes ſee the uterus ruptured at its fundus by the force of its own contraction, when there is at the collum uteri, or in the pelvis, an obſtacle ſufficiently powerful to reſiſt the exit of the fœtus. In a ſeries of experiments made by M. Chauſſier on the irritability and ſenſibility of animals, he has ſeen the cavities of the heart dilate, and become ruptured, almoſt inſtantaneouſly, whenever he ſtopped the circulation in the great arteries. Thus, for inſtance, he has obſerved that, in a living animal, if we paſs a ligature round the trunk of the aorta, or merely compreſs it with a pair of forceps, the left ventricle and auricle become torn. But he finds that, if the compreſſion is made on the trunk of the pulmonary artery, although the right auricle and ventricle become conſiderably diſtended, and the contractions of the heart are redoubled, while every fibre of it ſeems to palpitate, yet no rupture of the right cavities takes place, at leaſt he has not ſeen an inſtance of it in his experiments.

M. Chauſſier has favoured me with two other facts, which may ſerve as additional proofs that every thing that diminiſhes the diameter of the aorta may occaſion a dilatation, and rupture, of the left cavities of the heart. The firſt of theſe facts is founded on the caſe of a man who died ſuddenly, in the year 1771, in conſequence of a diſpute with one of his companions. On diſſection, the parietes of the left ventricle were found to be thin and dilated, and at the apex there was an oblong rupture of about an inch in extent. Near the curvature of the aorta, M. Chauſſier diſcovered a tumour as large as a fiſt, and almoſt as hard as cartilage, which ſurrounded the trunk of the aorta a little below the origin of the ſubclavian and carotid arteries. The diameter of the aorta, at this part, was ſo diminiſhed by the compreſſion of the tumour, that the end of the little finger could not be introduced into it without difficulty.

The other fact is as follows. A young woman after the diſappearance of a cutaneous eruption had been ſubject to a variety of complaints, ſuch as pains of her limbs, head-ach, and vertigo; but the moſt diſtreſſing were a ſenſe of weight about the region of the heart, a great languor, difficulty of breathing, and at times very violent palpitations. Theſe ſymptoms were aſcribed to a ſuſpenſion of the menſes. Every remedy was uſeleſs, and at length in the year 1774, after ſhe had been ill three years, ſhe died ſuddenly while ſhe was coughing up blood. She was then in her twenty-firſt year.

On opening the body, M. Chauſſier found the left cavity of the cheſt full of blood, and the heart of an extraordinary bulk; the pericardium was ſo ſtrongly adherent to this viſcus that it could not be detached from it; the left auricle and ventricle were ſo much dilated as to admit eaſily the two fiſts, and their parietes were ſo thin that it was difficult to diſtinguiſh them from the pericardium. The dilatation, however, was not confined to the cavities of the heart, as the pulmonary veins were equally diſtended, and a rupture was diſcovered in the pulmonary vein that returns from the left lobe. This rupture was about nine tenths of an inch in extent, and was near the entrance of the vein into the lungs. The aorta was of its natural diameter, but the ſigmoidal valves were indurated and thickened, and as large as a ſmall almond, containing within their texture a thick white white ſubſtance. This alteration of ſtructure had impeded the free paſſage of the blood, and gradually produced the appearances obſerved by M. Chauſſier in the left auricle and ventricle.

